# Improving Sewer Damage Inspection: Development of a Deep Learning Integration Concept for a Multi-Sensor System

**DOI:** 10.3390/s24237786

**Published:** 2024-12-05

**Authors:** Jan Thomas Jung, Alexander Reiterer

**Affiliations:** 1 Department of Sustainable Systems Engineering, University of Freiburg, Georges-Köhler-Allee 10, 79110 Freiburg im Breisgau, Germany; alexander.reiterer@ipm.fraunhofer.de; 2 Fraunhofer Institute for Physical Measurement Techniques IPM, Georges-Köhler-Allee 301, 79110 Freiburg im Breisgau, Germany

**Keywords:** automated inspection, damage detection, sewer pipes, artificial intelligence, robotic inspection, computer vision, urban infrastructure, 3D vision, point cloud, LiDAR

## Abstract

The maintenance and inspection of sewer pipes are essential to urban infrastructure but remain predominantly manual, resource-intensive, and prone to human error. Advancements in artificial intelligence (AI) and computer vision offer significant potential to automate sewer inspections, improving reliability and reducing costs. However, the existing vision-based inspection robots fail to provide data quality sufficient for training reliable deep learning (DL) models. To address these limitations, we propose a novel multi-sensor robotic system coupled with a DL integration concept. Following a comprehensive review of the current 2D (image) and 3D (point cloud) sewage pipe inspection methods, we identify key limitations and propose a system incorporating a camera array, front camera, and LiDAR sensor to optimise surface capture and enhance data quality. Damage types are assigned to the sensor best suited for their detection and quantification, while tailored DL models are proposed for each sensor type to maximise performance. This approach enables the optimal detection and processing of relevant damage types, achieving higher accuracy for each compared to single-sensor systems.

## 1. Introduction

### 1.1. Background

The foundation of urban existence relies on the efficient removal of sewage to prevent the spread of diseases and pests. Although largely invisible and therefore often overlooked by the general public, sewer systems are considered critical infrastructure because of their essential role. Consequently, they require regular inspection and maintenance, as in Germany, for example, in intervals of 10 to 15 years [1,2] or as in France every 10 years [3].

This situation leads to two significant challenges. First, the inspection of each section every 15 years is a monumental task, given the vast scale of the German sewer network, which spanned 608,000 km in 2019 [4]. These inspections are highly manual processes that require substantial personnel, thereby imposing a significant financial burden on municipalities. Second, the 15-year interval between inspections poses considerable risks. If a minor defect is overlooked during an inspection, it remains undetected for several years and could develop into a major issue, impairing the functionality of the sewer system and necessitating much more costly repairs. Additionally, the manual nature of the inspections and the variability in assessments by different inspectors complicate the reliability and consistency of evaluating the condition of the sewer system. To automate this process and enhance both cost-efficiency and reliability, extensive research has been conducted over the past several years in the field of autonomous sewer inspection, driven by both scientific and industrial interests. Significant improvements in autonomous sewer inspection models have been made possible by advancements in AI, particularly in the domain of computer vision. However, the diversity of methods and objectives pursued by researchers in this field complicates the ability to consolidate a cohesive understanding of the current research.

### 1.2. Current Inspection Workflow

The inspection of a sewer system can be divided into the processes of traversal, individual damage assessment, and overall evaluation. Typically, the traversal is conducted using a robotic platform equipped with a closed-circuit television (CCTV) camera. This robotic platform, which is connected to the inspection vehicle, provides the inspector with a live video feed from the camera, which is usually additionally recorded. During the inspection, the individual damage assessment is performed by the inspector, who navigates the movable camera head on the wheeled platform to capture images of the damage and assigns a corresponding evaluation. The processes of traversal and damage assessment are therefore integrated, requiring the damage assessment to be performed under time constraints, as the section of the sewer system and the overlying road remain blocked during this time. Subsequently, an automated overall evaluation of the sewer is conducted based on the individual damage assessments.

**Figure 1:**
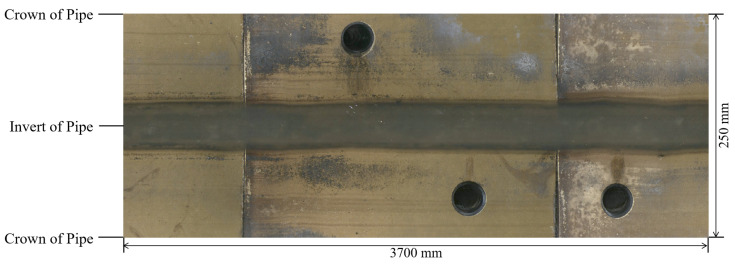
Unfolded 2D representation of the pipe surface, facilitating enhanced examination of potential pipeline damages. The composite image was generated from consecutive frames captured by a fisheye camera.

Recent inspection systems [5] employ a fish-eye camera in place of the traditional pan-and-tilt camera, thereby capturing the entire circumference of the pipe in a defined distance interval. This innovation enables fully automated data acquisition and subsequent damage assessments, thus facilitating true automation in damage evaluation. Moreover, from these individual images, an unfolded representation of the pipe can be computed, as illustrated in Figure 1, offering new opportunities for damage inspection and the potential application of AI.

### 1.3. Defect Types in Sewer Pipes

Sewer pipes are constructed from a variety of materials, each presenting distinct characteristics and potential vulnerabilities. Historically, masonry was a common material in the construction of sewer systems in Germany. In recent decades, however, concrete and stone have emerged as the dominant materials in modern sewer infrastructure, accounting for approximately 40% and 30% of the total, respectively. More recently, the use of polyvinylchlorid (PVC) has been rising, now accounting for nearly 20% of all sewer pipes. While each of these materials is prone to different types of damages, they are categorised across all materials in the unified assessment system, defined by the DIN EN 13508-2 standard [6], listed in Table 1, used as standard for sewer inspections within the European Union.

Next to the European assessment system, other prominent yet similar coding standards are used in North America (PACP), the United Kingdom (MSCC), and Australia (WSA 05), which generally align in terms of the types of defects they aim to detect. Beyond the categorisation of defects—of which there are 23 categories in the European DIN standard—the severity of each defect must also be assessed. The European system requires a detailed description of the defects, upon which the severity grade is subsequently determined. The requirements for these additional defect descriptions, detailed in Table 1 under “Characterization” and “Additional Information”, vary depending on the type of defect and can range from identifying the root type in cases of root intrusion to providing a detailed description of cracks, including the crack width. For fully automated damage detection, these fine-grained damage assessments are particularly relevant.

### 1.4. Recent Review Papers

The field of automated sewer inspection has its origins dating back to the year 2000, with the application of a back-propagation algorithm by Moselhi et al. [7] for the classification of damages in sewer pipes. However, since the late 2010s, traditional machine learning algorithms have largely been superseded by DL models, which have demonstrated significantly superior performance. Li et al. [8] provide a comprehensive overview of both classical machine learning algorithms and DL models in image-based defect inspection up until early 2022. Similarly, Rayhana et al. [9] offer a thorough review of hardware and software solutions for addressing the challenges of automated sewer inspection. Moradi et al. [10] compare various sensor systems used in sewer inspection, offering an in-depth analysis of the strengths and weaknesses of these technologies. Furthermore, their work provides a detailed assessment of the advancements and challenges associated with image-based automation techniques in sewer inspection. The most recent review of image-based automation methods in this domain is provided by Sun et al. [11], who examine various DL models used in image-based automation.

### 1.5. Contribution

This paper contributes to the field of automated sewer inspection by proposing a multi-sensor system concept coupled with a DL integration concept designed to facilitate the efficient adaptation of DL models to the data outputs generated by the system. The sensor system addresses the limitations currently inherent in the data produced by inspection robots, which often hinder the training and performance of DL models. To investigate and understand these limitations, an extensive review of the existing DL approaches applied to images from sewer pipe inspections is conducted, with a specific focus on the data used for training to identify weaknesses (Section 2).

In addition, the emerging research area of utilising point clouds for sewer pipe inspections is examined to uncover potential applications and opportunities (Section 3). Based on the insights gained from both the review of the existing DL approaches and the exploration of point cloud applications, a multi-sensor system concept is proposed, comprising three sensors: a front-facing camera for detecting damages within the pipe, a high-resolution camera array for identifying damages present on the pipe surface, and a LiDAR sensor for detecting structural and geometric damages in point clouds, enabling precise quantification of these defects (Section 4).

To complement the sensor system, damage classes are systematically assigned to the sensor best suited to detect each specific type of damage. Furthermore, a DL integration concept is developed that leverages DL models tailored to the unique data outputs of the proposed sensor system.

### 1.6. Review Methodology

This literature review focuses on publications from the beginning of 2021 onward. Earlier works that utilise traditional machine learning concepts are irrelevant to the current state of the art and, therefore, excluded from this study. Li et al. [8] already provide a comprehensive overview of these earlier approaches. In addition to the predominantly image-based models that have been extensively reviewed in previous papers, our study also offers an in-depth analysis of the emerging research field of automated damage detection based on point clouds. For both domains, a comprehensive literature search was performed. The relevant papers were identified using Google Scholar with specific keywords. For image-based methods, the keywords (“Sewer” OR “Sewage” OR “Pipe”) AND (“Defect” OR “Damage” OR “Assessment” OR “Deep Learning”) were used. Given the relatively nascent stage of point-cloud-based methods, a broader search was conducted to avoid overlooking any relevant publications. The keywords for this search were (“Sewer” OR “Sewage” OR “Pipe”) AND “Point Cloud.” Following an initial relevance screening based on paper titles, a more detailed assessment was conducted by reviewing the abstracts. Subsequently a cluster of selected papers was created using ResearchRabbit, which further identified additional relevant papers through citation analysis that were not captured in the initial keyword search. In total, 31 papers on image-based damage detection and 11 papers on point-cloud-based damage detection were selected for this literature analysis.

## 2. Deep Learning Models for Image-Based Sewer Inspection

**Figure 2:**
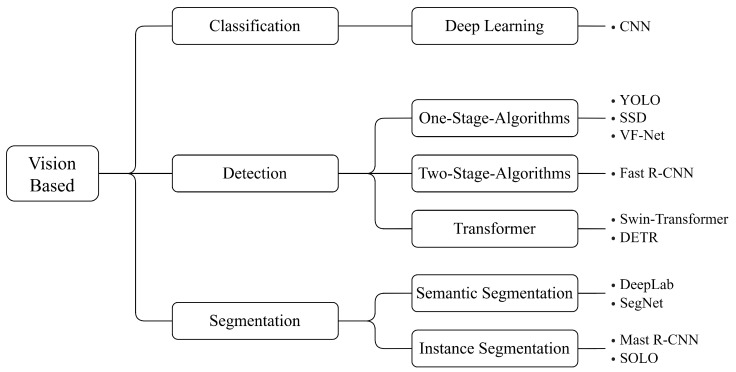
Overview of the primary tasks and the main algorithmic approaches employed in vision-based automated sewer inspection systems.

Although each paper addresses a distinct research topic, the foundational algorithms employed in these approaches are often well-established models. Figure 2 provides an overview of the commonly utilised methods and algorithms for the core tasks of damage classification, detection, and segmentation, which are elaborated upon in Section 2.5, Section 2.6 and Section 2.7, respectively. A comprehensive summary of all papers discussed in this work, including their respective goals, is provided in Table A1 in the Appendix A.

### 2.1. Datasets

A major challenge in creating and utilising datasets for sewer inspection is the significant manual effort required for data annotation. Although a substantial collection of damage imagery exists from previous sewer inspections, these images have only been classified but lack the necessary annotations for damage detection or segmentation within the images themselves. Such annotations are necessary for achieving more accurate damage assessment. The annotation process is labor-intensive and time-consuming, as it requires precise identification and labelling of damage within each image. To mitigate this burden, data augmentation techniques can artificially expand the existing datasets by altering images through processes such as rotation and scaling, thereby reducing the reliance on extensive manual annotations. Consequently, many studies [12,13,14,15,16,17,18,19,20,21,22,23,24,25,26,27,28,29] employ data augmentation methods.

The annotation process is labor-intensive and time-consuming, requiring precise identification and labelling of damage within each image. These high costs and efforts often discourage the publication of datasets, leading to a scarcity of publicly available data. Moreover, the limited availability of such datasets is further constrained by privacy and security concerns, as sewers are considered critical infrastructure. Notwithstanding these challenges, a limited number of public datasets have been made available in recent years. The Sewer-ML dataset [30], released in 2021, represents the most extensive public dataset of its kind. It is a comprehensive, publicly accessible multi-label sewer defect classification dataset, comprising 1.3 million images. These images were collected from 75,618 sewer inspection videos conducted over a nine-year period (2011–2019) by professional inspectors from three Danish utility companies. The dataset is annotated according to the Danish sewer inspection standard, which encompasses 18 specific defect classes, including cracks, breaks, deformations, displaced joints, infiltration, roots, and various types of deposits. Each defect is associated with a class-importance weight (CIW) that reflects its economic impact. The dataset captures a broad spectrum of sewer pipe conditions, encompassing both main and lateral pipes, and includes diverse materials, shapes, and dimensions, thereby rendering it representative of real-world sewer inspections. In 2022, a dataset comprising sewer inspection videos, as opposed to images, was published and introduced in the VideoPipe Challenge [31]. This challenge is centred around two distinct datasets: QV-Pipe and CCTV-Pipe, each designed to address specific tasks in urban sewer system inspection. The QV-Pipe dataset is used for the task of video defect classification and contains 9601 short video clips, with a total duration exceeding 55 h. These videos capture various pipe anomalies and are annotated with multiple labels, representing 17 different categories. On the other hand, the CCTV-Pipe dataset is intended for temporal defect localisation. It comprises 575 longer, untrimmed videos, totalling 87 h, each focusing on structural and functional defects within the pipes. Unlike QV-Pipe, the continuous nature of these videos requires the detection of defects and identification of their exact timing within the extended video duration. Another smaller public dataset for damage detection is the storm drain dataset [32], comprising 1000 annotated damage images in storm drains, categorised into six distinct defect classes. A dataset facilitating both damage detection and segmentation has been introduced by Y. Li et al. [27]. Their collection comprises nearly 4000 high-resolution images captured from CCTV inspections of underground sewers in Seoul, South Korea and includes manually annotated images covering six prevalent defect types: open joint, faulty joint, protruding lateral, crack, broken pipe, and surface damage. The dataset is divided into 3000 images for training—expanded to 9000 images through data augmentation—and around 1000 images for testing, offering a comprehensive resource and serving as a benchmark for comparing different approaches. Table 2 summarises all public datasets.

**Figure 3:**
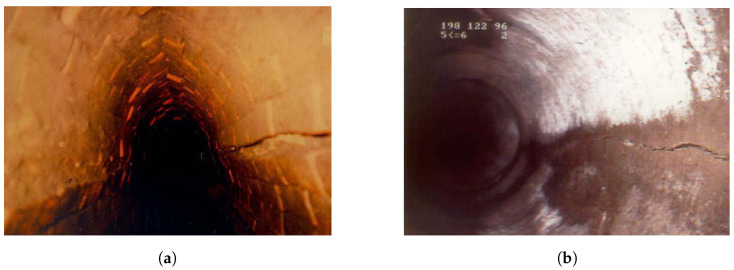
The damage coding of two actual inspections according to and from DIN EN 13508-2 [6]. Image (**a**) illustrates the challenge of estimating the cross-sectional reduction of the canal based solely on a single image. Image (**b**) highlights the issue of inconsistent documentation of a damage event compared to the damages that are actually visible in the image. It is important to note that the low resolution of the images often hinders the clear visibility of certain damages. (**a**) Pipe with a longitudinal crack at the 3 o’clock position and a 15% reduction in cross-section; (**b**) Two cracks are noted in the inspection report at the 3 and 4 o’clock positions on the pipe’s circumference, though only one is visible in this image.

The datasets utilised in this study are derived from historical records and documentation of prior inspections. These damage assessments span several decades, involving a range of camera technologies and different inspectors. The resulting variability in the data poses substantial challenges for training an AI model. Figure 3 presents the documentation of two example damages. In Figure 3a, alongside the evident crack on the right side, a cross-sectional reduction of 15% is noted, as estimated by the inspector. Retrospective estimation of this reduction based on a single image, without additional information about the original pipe geometry, is unreliable. In Figure 3b, the inspector identified two cracks at the 3 o’clock and 4 o’clock positions, although only one crack is distinctly visible. Such inconsistencies and errors in documentation further compromise the quality of the datasets.

In the work of X. Wang et al. [33], they discussed the occasional lack of clear distinction between photos with and without damage. The high similarity between images of defective and non-defective instances increases the susceptibility of AI models to misclassification. Biswas et al. [34] observed a significant performance decline due to low inter-class variability when transitioning from a binary classification approach—where the model was trained to distinguish a single defect category from non-defective instances—to a one-vs-all classification strategy. In the one-vs-all approach, each defect category was compared against all other categories, including non-defective instances. This shift led to a decrease in average balanced accuracy by nearly 5%, primarily due to the low inter-class variability among several defect categories, which, at times, exhibited indistinguishable similarities.

As a future goal, it will be necessary to employ advanced imaging sensors that can better distinguish between different types of damages and to ensure more precise and consistent documentation practices in order to achieve reliable automated AI-based damage detection.

### 2.2. Image Preprocessing

To overcome the limitations from the low quality of the dataset images, effective preprocessing is crucial for accurate classification, detection, and segmentation. In the challenging environment of sewer pipes, where lighting conditions are poor and textures can be low-contrast or murky, these preprocessing techniques enhance image quality, reduce noise, and ensure that critical features, such as cracks, leaks, and blockages, are clearly visible for analysis.

Contrast enhancement techniques are vital in low-light environments to make subtle features more discernible. Several studies have employed dynamic histogram equalisation to enhance image contrast, improving the visibility of defects under challenging conditions. This method has been widely used, as seen in [12,14,24,35], to bring out details that would otherwise be obscured in dark or low-contrast areas. Brightness enhancement was also applied by [28], applying HSV adjustment, and in [20,36], though the specific methods used here were not detailed. Noise reduction techniques are equally important, as noise can obscure critical details in images captured within sewer pipes. Bilateral filters were used in [12] to reduce noise while preserving edges, ensuring that important structural features remain sharp. GCANet, a more advanced technique, was applied in [35] for noise reduction and in [27] for dehazing, highlighting its versatility in both noise and haze removal. Additionally, blurring was used in [14] to further reduce noise, albeit with a more straightforward approach, while a general denoising strategy without specified methods was mentioned in [20]. Dehazing and defogging techniques are essential in environments where images are often obscured by fog or haze. The dark channel prior method, utilised by [15], is a popular technique for defogging images, making them clearer for analysis. For dehazing, several studies employed different methods, such as DehazeFormer in [20], DehazeNet in [23], and GCANet in [27], all aimed at removing haze and improving image clarity. Saturation adjustment and sharpness enhancement were employed by [20,36] to make colours more vivid and enhance the sharpness of images, thereby improving the detectability of defects. HSV enhancement, applied by [37], adjusted the hue, saturation, and value of the images, making them more suitable for subsequent analysis. Morphological operations were used in [14] to clean up noise and improve the structural integrity of the features in the images. Finally, contrast enhancement through the mean-shift algorithm and moth swarm algorithm was explored by [23], further refining the image quality. Beyond enhancing image quality, some studies also focused on quality control methods to improve the dataset used for training. For example, ref. [13] deleted similar images to avoid redundancy, ensuring that the training data were diverse and informative. Similarly, low-quality images from larger datasets were deleted by [18] to ensure that only high-quality images were used for analysis, improving the robustness of the models trained on these datasets.

However, the use of preprocessing can be seen as a double-edged sword. While it improves image quality, it also alters the image in ways that may not reflect the real-world conditions under which new images are captured. This can lead to potential issues when models trained on preprocessed images encounter raw images with different lighting or environmental conditions where preprocessing cannot be equally performed. Moreover, in real-time damage detection scenarios, extensive preprocessing may not be feasible due to time constraints, which could limit the applicability of these techniques in practice.

### 2.3. Evaluation Metrics

The reviewed papers employ a range of evaluation metrics, each selected according to the specific objectives of the tasks under consideration. For image classification, commonly used metrics include accuracy (Acc), F1-Score (F1), and mean average precision (mAP). Accuracy quantifies the proportion of correctly classified images, providing a general measure of performance. However, in cases of imbalanced datasets, F1-Score is preferred, as it harmonises precision and recall, offering a more balanced evaluation. mAP is widely applied in multi-class classification to assess the model’s ability to balance precision across varying levels of recall.

In the context of damage detection, metrics primarily focus on evaluating both detection and localisation accuracy. mAP is commonly utilised to assess detection performance by averaging precision across recall levels at a fixed IoU (intersection over union) threshold, typically IoU@0.5, meaning that a prediction is deemed correct if the predicted and ground truth bounding boxes overlap by at least 50%. However, this single threshold may not fully capture the model’s localisation accuracy. To provide a more comprehensive evaluation, mAP@0.5:0.95 is often used, which averages precision over multiple IoU thresholds ranging from 0.5 to 0.95. This method offers a broader assessment of the model’s ability to detect and localise objects across varying levels of overlap. While less commonly employed, some studies also use IDF1, which measures the consistency of object identity tracking over time, and Top-1 accuracy, which evaluates whether the model’s highest-confidence prediction is correct. For segmentation tasks, different metrics are used depending on the type of segmentation. In instance segmentation tasks, IoU@0.5 and mAP are commonly employed to assess the accuracy of individual object instance segmentation and localisation. In contrast, for semantic segmentation, pixel-level metrics such as mean intersection over union (mIoU) and mean pixel accuracy (mPA) are used to evaluate the overlap between predicted segments and ground truth, providing an accurate measure of pixel-wise classification performance.

The metrics, along with their respective evaluation goals, are summarised in Table 3. Moreover, the lack of consistency in datasets—where many studies utilise proprietary data instead of publicly available benchmarks—combined with the use of different evaluation metrics as the papers address different goals, makes direct comparisons between the results of these papers extremely challenging. These variations in both datasets and evaluation methodologies significantly hinder the ability to draw meaningful conclusions regarding the relative performance of the models, which are therefore not drawn in this review.

### 2.4. Investigated Damage Types in Image-Based Research

The creation of a qualitative and clean dataset presents significant challenges, particularly due to the requirement for manual damage labelling. Additionally, there is a high degree of class imbalance, with some types of damage being scarcely represented in the datasets [30]. However, a critical quantity of images is necessary to ensure adequate representation. As a result, not all damage classes are consistently examined in the studies under review. Comparisons between these studies are complicated by the lack of a standardised classification system. Many papers do not employ official damage classes but instead select their own categories. To facilitate comparability, Table 4 organises the image-based damages according to the types of damages examined. These damages have been categorised according to the classes defined by the DIN Norm EN 13508-2 [6].

### 2.5. Damage Classification Methods

Classification is a fundamental task in sewer defect detection that involves categorising images into predefined classes based on their content. It can be considered the most straightforward task since the images are already classified by inspectors, eliminating the need for additional annotation. Biswas et al. [34] conducted a comprehensive evaluation of various state-of-the-art DL models to classify sewer defects accurately. The study involved three key experiments: single-defect classification, one-vs-all classification, and classification under weight pruning. They employed multiple neural network architectures, including HRNet, ResNet-152, MobileNet V3 Large, DenseNet-264, Inception V4, and EfficientNet. By optimising binary cross-entropy loss with strategic training regimens and hyper-parameter tuning, the models effectively handled highly imbalanced and noisy data. Additionally, weight pruning was implemented to create resource-efficient models, achieving significant compression with minimal performance loss. This thorough evaluation provided a solid foundation for understanding the capabilities and limitations of the existing models. X. Wang et al. [33] proposed the PIPE-CovNet model, which aims to detect surface abnormalities in wastewater pipe images by classifying all defective images from the Sewer-ML dataset [30] into one category and all non-defective images into another. The model leverages a novel architecture that combines two convolutional neural networks (CNNs) with different kernel sizes, capturing a broader range of features by balancing shape- and texture-based information. The training process is divided into two stages: initially, each CNN is trained separately, and their intermediate features are then concatenated for final training. This strategy, combined with dropout layers to prevent overfitting, aims to improve the robustness and performance of the model. Hu et al. [38] developed a trustworthy and robust classification system by segmenting inspection videos into different focal lengths using a focal segment module to capture multi-scale information. Each segment is classified with evidential DL, which also estimates prediction uncertainty. The classifications from different segments are then combined using a joint expert scheme for a more reliable final decision. An evidential disambiguation strategy further enhances reliability by resolving ambiguity in uncertain cases, particularly for unknown or rare defects. Table 5 summarises the investigated papers.

### 2.6. Damage Detection Methods

Detection plays a crucial role in the current landscape of AI applied to sewer inspections. While classification methods have made significant progress, they are not yet advanced enough to replace human inspectors entirely. As a result, AI efforts in this field are focused on augmenting manual inspections by improving detection capabilities. Damage detection, therefore, receives significant research attention, aiming to identify defects in real-time and with higher accuracy, ultimately reducing the workload on human inspectors and enhancing the reliability of inspections.

#### 2.6.1. YOLO

The you only look once (YOLO) algorithm, initially introduced in 2015 [46], revolutionised real-time object detection by predicting bounding boxes and class probabilities directly from full images in a single pass. Successive versions have brought numerous enhancements: YOLOv2 [47] introduced anchor boxes to improve accuracy, YOLOv3 [48] incorporated residual connections for better feature extraction, and YOLOv4 [49] added mosaic augmentation to enhance speed and performance. YOLOv5 [50], although not officially released by the original creators, gained popularity for its ease of use, enhanced speed, and flexibility in deployment, becoming widely adopted in various applications. YOLOv6 [51] further improved on these aspects by introducing efficient decoupled head architectures and optimisation techniques that significantly boosted both speed and accuracy, making it particularly effective for industrial and embedded applications. YOLOv7 and YOLOv8 introduced more efficient architectures and scaling techniques, further improving speed and accuracy, though they have not yet been applied in sewer inspection methods.

As YOLO performs detection in a single pass, compared to two-stage approaches like Faster R-CNN [52], predictions are processed faster, and it is therefore more efficient for real-time applications. This fundamental difference has driven various adaptations and enhancements to the YOLO framework for specific applications such as sewer defect detection. Tan et al. [13] improved YOLOv3 by incorporating advanced loss functions and adaptive anchor box calculations, enhancing bounding box accuracy and data robustness. They also integrated cross stage partial connections, spatial pyramid pooling, and path aggregation network to optimise feature extraction while reducing computational costs. Building on the YOLO framework, Situ et al. [42] used transfer learning with YOLOv2, leveraging pretrained models like AlexNet, ResNet, and Inceptionv3 to fine-tune on a sewer defect dataset, with Inceptionv3 achieving notable precision. Further extending this approach, Situ et al. [15] employed YOLOv5, incorporating transfer learning and channel pruning to enhance both accuracy and speed, making the model suitable for deployment on portable devices. Huang et al. [17] and Zhao et al. [18] both focused on enhancing YOLOv5 by integrating convolutional block attention modules (CBAM). Ref. [17] added CBAM to the backbone and neck of YOLOv5l6, while ref. [18] developed YOLOv5-Sewer, a lightweight model optimised with MobileNetV3 blocks and additional attention mechanisms, aiming for efficient detection on low-performance devices. T. Wang et al. [37] further refined YOLOv5 with involution modules, GSConv, and CBAM, targeting reduced parameters and improved feature detection in complex backgrounds. Similarly, Oh et al. [19] utilised an enhanced YOLOv5 with CBAM and added a micro-scale detection layer for better identification of small cracks. Lastly, Yin et al. [41] applied YOLOv3 in their system, automating defect detection in sewer pipe videos and generating detailed reports. This approach highlighted the versatility of YOLO models in processing video data for comprehensive defect analysis. Table 6 provides a summary of the reviewed papers focused on detection tasks, including YOLO, as well as other approaches.

#### 2.6.2. R-CNN, SSD, Transformer, and Custom Methods

The region-based convolutional neural network (R-CNN) [52] and its successors, Fast R-CNN [53] and Faster R-CNN [54], have been pivotal in advancing object detection. Introduced in 2014, R-CNN combined region proposals with convolutional neural networks to achieve high accuracy by generating around 2000 region proposals and processing each through a CNN. However, this approach was computationally intensive. Fast R-CNN, introduced in 2015, addressed these speed limitations by feeding the entire image into a CNN to produce a feature map, from which region of interest (RoI) pooling extracted fixed-size feature maps for classification and regression in a single pass, significantly improving detection speed and training efficiency. Faster R-CNN, introduced in 2016, integrated a region proposal network (RPN) into the CNN architecture to propose regions nearly for free, merging with Fast R-CNN into a single, unified network. This integration enabled simultaneous region proposal and object detection, greatly enhancing real-time detection capabilities.

Using the Faster R-CNN framework, Guo et al. [45] proposed an enhanced algorithm for detecting and classifying pipe defects integrated with a pipe-extended feature pyramid network (P-EFPN). This modified Faster R-CNN introduced as P-EFPN captures more detailed texture information, particularly in edge regions, and embedded a super-resolution (SR) module within the feature pyramid network to generate high-resolution features from low-resolution images. These enhancements aimed to address the limitations of existing methods that often lose critical texture details during the conversion of 3D pipe surfaces into 2D images, thereby improving the detection and classification accuracy of defects such as deformation, corrosion, and cracks.

Single shot multibox detector (SSD) is an object detection framework known for its balance between speed and accuracy. Unlike Faster R-CNN, which uses a two-stage approach involving region proposals and classification, SSD performs both tasks in a single pass, making it faster and more efficient for real-time applications. Shen et al. [43] developed the enhanced feature extraction single shot multibox detector (EFE-SSD) to improve sewer pipeline defect detection. Their model includes the receptive field block (RFB) for enhanced multi-scale feature extraction, a skip densely connected module for better small defect detection, and an efficient channel attention mechanism to focus on relevant features. Additionally, they replaced cross-entropy loss with focal loss to address class imbalance and improve detection accuracy.

Building on the idea of improving feature extraction, Yu et al. [20] introduced a novel approach using a composite backbone with Swin Transformers for sewer defect detection. This model leverages both an assisting and a lead backbone for comprehensive feature extraction and incorporates Cascade R-CNN to refine predictions through multiple stages, capturing both global and local features. Similarly, Dang et al. [35] adopted a transformer-based model (DETR) but employed an encoder–decoder architecture to directly predict defect locations and bounding boxes. Their method incorporates complete intersection over union (CIoU) for precise bounding box calculations and employs multi-head attention mechanisms to assess the severity of detected defects, streamlining the detection process by eliminating the need for traditional object detection components like anchor generation. In contrast, Y. Li et al. [16] proposed Pipe-VFNet, which enhances sewer defect detection by using an advanced ResNeSt backbone along with a coordinate split attention module. Their model integrates an attention-path aggregation feature pyramid network (Att-PAFPN) to combine path aggregation with attention mechanisms and employs efficient focal loss (EFL) to address data imbalance. On a related note, Dang et al. [14] utilised a modified VGG-19 network for sewer defect detection, enhancing it with additional convolutional layers, batch normalisation, cost-sensitive learning, and ensemble methods like XGBoost and LightGBM. They further incorporated explainable AI techniques, such as class activation mapping (CAM), to interpret predictions. Yusuf et al. [28] evaluated VGG19 in comparison with other models, including AlexNet, VGG16, and EfficientNet, ultimately determining that VGG19 provided the most effective performance for encrustation detection. To tailor VGG19 to this specific task, they reduced its depth, thereby balancing computational efficiency with robust feature extraction capabilities, which proved particularly advantageous for identifying subtle defects. M. Wang et al. [44] developed an innovative algorithm for automating defect tracking in sewer pipelines. Their system integrates DL for defect detection, a metric learning model for feature extraction and analysis, and a Kalman filter for predicting defect trajectories. The Hungarian algorithm is used to associate predictions across frames, considering motion, appearance, and defect class distances, effectively tracking defects over time. Ma et al. [36] take a different approach by integrating inception and residual networks into a fusion CNN. Their method is bolstered by StyleGAN-SDM, which generates synthetic images to expand the dataset, addressing the challenge of limited training data. This fusion network combines the feature extraction capabilities of the inception module with the robustness of the residual module. Similarly, D. Li et al. [12] presented an advanced algorithm that integrates local and global features, using a strengthened region proposal network (SRPN) and a fine-grained classification network, fine-tuned with a pre-trained VGG16. Lastly, Yang et al. [21] developed a weakly supervised collaborative localisation network for sewer pipe defect detection, which integrates three main components: an attention refinement module (ARM) to extract richer semantic features, a collaborative localisation module (CLM) to enhance object region localisation by combining a localisation and segmentation branch, and an image iteration module (IIM) to iteratively refine the detection by covering the complete object area. This approach allows for accurate defect classification and localisation without the need for pixel-level annotations.

### 2.7. Damage Segmentation Methods

Segmentation is a fundamental technique in computer vision, crucial for analysing and interpreting images by dividing them into meaningful parts—in this case, extracting damaged areas to better describe and quantify them. This chapter details the primary segmentation methods, namely, semantic and instance segmentation, discussing their different methodologies and outcomes.

#### 2.7.1. Semantic Segmentation

Semantic segmentation on images is a computer vision technique that involves classifying each pixel in an image into a predefined category. The goal is to partition the image into regions based on the categories of objects they represent without differentiating between different instances of the same class. Semantic segmentation is widely used in applications where the distinction between different instances of the same class is not as relevant as scene understanding. In the context of sewer pipe inspection, the potential occurrence of similar damages within a single image is typically rare but cannot be entirely excluded. Therefore, the applicability of semantic segmentation in such scenarios must be carefully considered.

A state-of-the-art DL model designed for semantic image segmentation is DeepLabv3+ [55], building upon the capabilities of its predecessor, DeepLabv3 [56]. DeepLabv3+ introduces a simple yet effective decoder module on top of the encoder that uses atrous convolutions and atrous spatial pyramid pooling (ASPP). The encoder captures rich semantic information, while the decoder refines the segmentation results, particularly along object boundaries. The input image is processed though a backbone network (e.g. ResNet), followed by ASPP and a decoder, combining features from ASPP and the low-level features from the encoder. The final result is a segmentation map that classifies each pixel into a specific class. Both Zhou et al. [22] and Dang et al. [23] used DeepLabv3+ with different modifications. Zhou [22] tested various backbone networks and found ResNet50 to be the most effective. Dang [23] used a deeper ResNet152 backbone, helping to capture more high-level features. To optimise processing efficiency, they implement a frame reduction algorithm that selects only frames containing potential defects, thereby reducing computational load while maintaining high segmentation accuracy. Another deep-learning semantic segmentation model is SegNet, used by He et al. [24]. SegNet is a DL architecture designed specifically for semantic pixel-wise segmentation, often used in applications requiring detailed classification of image pixels into different categories and is notable for its efficiency and effectiveness in segmentation tasks. While the encoder extracts high-level features, the decoder uses instead of traditional upsampling methods the pooling indices from the corresponding encoder layers. This helps in preserving spatial information. He et al. utilises a Deep-CNN based on the Segnet architecture. M. Wang et al. [40] uses a DilaSeg model, combined with a refinement with conditional random fields (CRF). The DilaSeg model employs dilated convolutions for initial segmentation. The dilated convolutions are used to increase the receptive field of the layers, without increasing the number of parameters, allowing the model to capture more information from the image, helping especially in conditions with complex backgrounds. Applying CRFs refines the initial segmentation masks by using probabilistic models to find spacial and contextual relationships between pixels. M. Li et al. [29] propose PipeTransUNet, a fusion model combining CNNs and Transformers, based on the U-Net architecture [57]. The model leverages ResNet50 for fine-grained feature extraction, CBAM to enhance attention to critical spatial and channel-specific features, and a Transformer encoder to capture both local and global contextual information, addressing U-Net’s limitations with intricate defect boundaries. Severity ratings are computed based on pixel proportions and defect-specific measurements, adhering to established standards to ensure accuracy and interpretability.

#### 2.7.2. Instance Segmentation

**Figure 4:**
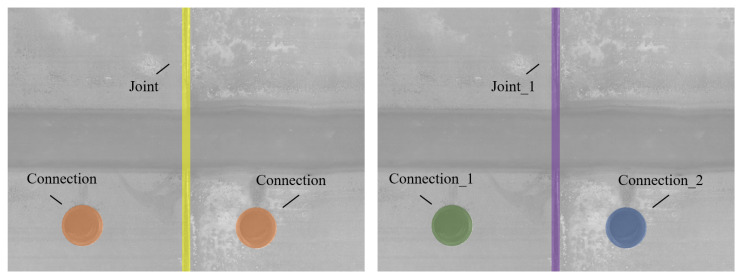
Difference between semantic segmentation (**left**) and instance segmentation (**right**), based on the pipe surface image from Figure 1. Instance segmentation distinguishes between individual instances of the same category, allowing for the identification of multiple damages of the same type in a single image.

Instance segmentation is a sophisticated computer vision task that not only classifies each pixel into a category but also distinguishes between different instances of the same category, as visualised in Figure 4. The goal is to provide a detailed understanding of the scene by identifying and segmenting each individual object separately. This level of detail is crucial in applications where it is important to identify and differentiate between multiple objects of the same class, such as in robotics, surveillance, and advanced autonomous driving systems.

Mask R-CNN [58] is a widely used DL framework designed for object instance segmentation. It extends the Faster R-CNN model by adding a branch that predicts segmentation masks for each region of interest (RoI). Mask R-CNN starts with an input image and utilises a region proposal network (RPN) to generate candidate regions (RoIs) where objects might be located. To ensure accurate feature mapping, each candidate region undergoes precise alignment through a process called RoIAlign. For each RoI, the network predicts the class of the object, refines its bounding box, and simultaneously generates a binary mask that identifies the specific pixels belonging to the object within the bounding box. Rayhana et al. [25] proposed the CSA-MaskC-RCNN algorithm, which incorporates several modifications to the standard Mask R-CNN to enhance its performance in defect segmentation. These modifications include the channel-spatial attention (CSA) mechanism and an additional canny edge detection (CED) branch. The CSA mechanism improves feature extraction by concentrating on significant regions within the input images. This is achieved through the sequential application of channel attention, which prioritises key feature maps, and spatial attention, which emphasises important spatial locations. Additionally, the integration of a CED branch refines defect boundaries, leading to more accurate segmentation outcomes. Fang et al. [26] also proposed enhancements to the Mask R-CNN architecture to improve its effectiveness in defect segmentation. Their improved Mask R-CNN integrates a split attention module and a balanced L1 loss module. The split attention module helps in retaining effective feature representations while discarding redundant features, thereby improving the model’s overall performance. Additionally, the balanced L1 loss function enhances object localisation performance by balancing the contribution of inliers and outliers during training, resulting in more accurate and robust defect detection and segmentation. Y. Li et al. [27] introduces an advanced instance segmentation algorithm named Pipe-SOLO. The Pipe-SOLO model incorporates a robust backbone structure known as Res2Net-Mish-BN-101, which enhances feature extraction through multi-scale representation and better generalisation by replacing the traditional ReLU activation function with Mish. Additionally, the model employs an enhanced bi-directional feature pyramid network (EBiFPN) for improved feature fusion across different levels of the network, facilitating more accurate defect localisation. The segmentation head utilises dynamic convolution kernels to streamline computational complexity and implements a novel matrix non-maximum suppression algorithm to optimise mask selection. To enhance comparability, they made their dataset publicly available, allowing different approaches to be compared. Table 7 provides a summary of the reviewed papers focused on segmentation tasks.

### 2.8. Discussion

In recent years, vision-based damage inspection methods have advanced considerably, largely driven by the integration of DL models. Research in this field can generally be categorised into three areas: image, thereby damage, classification, damage detection, and damage segmentation, with the greatest focus on damage detection and relatively few studies on purely image classification. Although each research area faces unique challenges, several common issues affect all three. Image classification models often contend with highly inconsistent datasets. Depending on the classification system used, around 20 different damage categories need to be differentiated. The subtle distinctions between certain damage types, however, lead to low inter-class variability, complicating accurate classification. Furthermore, these minor differences often result in incorrect labelling by human inspectors, compromising dataset quality. Finally the scarcity of certain damage types also leads to a heavily imbalanced dataset. Treating damage identification only as a binary classification problem—simply detecting the presence or absence of damage—improves performance [34], as these dataset problems do not have such a large influence any more. However, this approach does not align with the goal of creating a comprehensive automated damage classification system. Avoiding these challenges, research in damage detection often narrows the scope by focusing on a subset of damage types that are both common and visually distinct, such as cracks, displaced joints, and root intrusions. However, this does not entirely overcome the issue of poor image quality, which remains a significant obstacle [17,42]. Low-resolution images, homogeneous textures, and low-light environments yield minimal features, creating particular difficulties for damage detection models. As these models are often designed to facilitate real-time human inspection, they are generally lightweight and struggle with low-contrast images in challenging inspection environments. Additionally, the models’ performance across the full range of damage categories remains unclear, which is critical to create a functional use case for these models. Damage segmentation models, however, show promising results due to their more complex architectures, with improved performance and enhanced feature extraction, even for small details. Among these, instance and semantic segmentation models dominate, with instance segmentation offering an advantage: it can distinguish between multiple occurrences of the same damage type within a single image. Segmenting at the pixel level also enables quantification of damages, providing an estimate of damage severity and further moving toward fully automated inspections. Nevertheless, similar to damage detection, most segmentation models are trained on datasets with limited damage categories, raising questions about their efficacy when applied to comprehensive datasets. The labour-intensive process of manually labelling damage types further compounds this challenge.

Despite these individual challenges, a shared issue across all research areas is the inherent difficulty of working with a challenging dataset. Damage images have historically been captured with low-resolution cameras under poor lighting, and damages are often partially cut off due to limited camera field of view. This situation suggests two possible solutions. First, further improvements in model capabilities can be pursued. Additional damage images can be labelled, data can be augmented and preprocessed, damage instances can be tracked across frames to boost model confidence, and model architectures can be refined. However, dataset quality sets an upper bound on accuracy, and this limit may soon be reached given the current data constraints. The second solution involves enhancing data quality, which would improve feature detection and reduce inter-class variability. Using multiple fixed cameras, rather than a single rotatable one, could enable comprehensive inspection of the pipe surface while providing consistent image perspectives, thereby reducing model complexity.

Finally, even though several studies use the same base architecture, such as the YOLO framework, meaningful comparison is hampered by differences in dataset size, damage types, and conditions studied, limiting cross-study insights. This lack of standardised comparison hinders progress in the field, as models and methods cannot be reliably assessed against each other, making it difficult to discern their relative strengths. Therefore, a public dataset, serving as a benchmark in damage detection and segmentation, is needed to make future research in this field comparable.

## 3. Potential of Point Clouds for Automated Sewer Inspection

The research on point-cloud-based damage detection methods is still in its early stages, particularly when compared to the more advanced image-based techniques. While representing sewer surfaces as point clouds offers great potential for damage detection, it requires significant preparatory work to obtain clean and usable data. The generation of point clouds within sewer environments can be achieved through various sensor technologies, as detailed by Bahnsen et al. [59]. Their study provides a comprehensive review of 3D modelling techniques, including both passive and active stereo vision, depth cameras, and LiDAR. Stereo vision, whether passive or active, utilises two cameras to capture images from different angles, with depth information calculated from the disparities between these images. Active stereo vision improves surface detail in low-texture environments, such as sewer pipes, by projecting a structured light pattern. However, the need for two cameras limits the application of stereo vision in narrow pipes, where space constraints present a significant challenge. Both depth cameras and LiDAR utilise time-of-flight (ToF) technology, measuring the time it takes for light to travel to a surface and return. Although they are based on the same underlying principle, their outputs and operational ranges differ. Depth cameras use infrared light to capture 3D depth information, typically stored as a depth map or a point cloud, while also providing a corresponding image, either in infrared or colour. LiDAR, in contrast, uses laser pulses, generally in the near-infrared spectrum, to generate point clouds without producing a conventional image. Nevertheless, LiDAR-generated point clouds can be fused with imagery from other sensors to augment both the spatial and visual data. Despite these distinctions, both LiDAR-based and depth-camera-based technologies are highly effective in generating precise 3D point clouds with a single sensor, from which a continuous 3D model can subsequently be derived. Finally, it should be noted that as both technologies employ near-infrared (NIR) light for distance measurement, this wavelength does not penetrate water; thus, as with vision-based inspection, it is essential to ensure that pipes are free from water during scanning.

### 3.1. Damage Detection Techniques

Using multi-view stereo vision, Ma et al. [60] proposed a method for reconstructing 3D models of potholes within concrete pipes and accurately quantifying their dimensions. The study measured potholes with maximum depths ranging from approximately 14 mm to 30 mm and areas spanning 12,400 mm² to 51,000 mm². The method achieved results with errors of 9.5% in maximum depths, 13.2% in mean depths, and 6.7% in areas. These findings demonstrate notable advancements over conventional structure-from-motion (SFM) techniques, reducing error rates by 19% to 25%. Their approach involved cylinder fitting through the random sample consensus (RANSAC) algorithm, coupled with boundary search techniques, to accurately quantify the geometric properties of defects. This process relied on generating a dense point cloud from multiple overlapping images, allowing for the construction of a cylindrical model that serves as a reference for isolating and measuring the dimensions of the potholes. While this method demonstrates significant potential for detailed 3D reconstruction, it remains questionable whether such a camera setup, requiring multiple cameras and specific distances between them, can be implemented on small robots designed for pipes with limited diameters. Using an RGB-D sensor, Pang et al. [61] also introduced an advanced method for the 3D reconstruction and quantification of potholes in concrete pipes. After preprocessing their point cloud data to reduce noise, they also used the RANSAC algorithm to precisely extract the cylindrical geometry of the pipes and isolate the damaged regions. Finally, an alpha shape algorithm was employed to reconstruct the surface of the potholes, enabling accurate measurement of their dimensions and volume. For internal damages, the method achieved an average relative error of 5.22%, with the volumes of the tested damages ranging from 155 cm³ to 265 cm³, demonstrating its potential for precise damage quantification. However, it was found that tilting the sensor towards the surface reduces the point cloud density, thereby decreasing the accuracy of quantification. This highlights the importance of maintaining a dense point cloud for precise damage assessment.

Beyond the investigation of holes and spallings, geometric deformations in sewer pipes have also been examined using point clouds. Li et al. [62] aimed to detect and quantify pipeline damages and deformations through a three-step process: first, preprocessing the point cloud data by aligning the pipeline axis and segmenting the data into 2D slices; second, meticulously extracting geometric features from these slices using least-median-of-squares (LMedS) fitting and density-based spatial clustering of applications with noise (DBSCAN) [63] clustering, effectively isolating the true pipeline geometry from noise and outliers; and finally, constructing a 3D geometric digital twin through density-based region growing, from which structural deformations in the pipes surface such as ovality and blockages are able to be identified. Ebrahimi et al. [64] also focused on measuring deviations from the norm in the pipeline surface due to erosion. Using a LiDAR system, they captured high-resolution 3D point clouds of the pipe interiors, providing detailed geometric data. To enhance measurement accuracy, preprocessing was applied to the raw point cloud data, filtering out noise caused by sewer flow and scanner errors. Erosion was then assessed by comparing the actual inner surface geometry, derived from the 3D model, with the nominal pipe radius, where deviations indicated the extent of concrete loss.

More extensive structural deviations have been investigated by Lin et al. [65], who proposed a novel method for detecting and assessing tunnel defects using 3D longitudinal deformation curves (3D-LDC) derived from high-resolution point cloud data obtained through LiDAR. This method involves segmenting the tunnel into individual sections, fitting cylindrical models to each segment using the RANSAC algorithm to accurately capture the tunnel’s geometry, and subsequently calculating the displacements, rotations, and joint dislocations between adjacent segments. These calculated deformation indices are then analysed to assess the structural integrity of the tunnel, enabling the identification of potential defects such as misalignment, rotation, and shearing much better than by current camera-based inspection methods.

### 3.2. AI-Driven Damage Detection

In all cited studies [60,61,62,64,65], methods have been developed to visualise damage in sewer pipelines. However, the assessment of the damage itself still predominantly occurs manually. Lin et al. [65] highlight in their work the lack of sufficient data to adequately validate and further develop their method, a challenge that can be seen as a general obstacle to the advancement of AI models in the use of point clouds for sewer inspection. The generation of a dataset large enough for research purposes is severely hampered by the limited accessibility of sewer environments. Henriksen et al. [66] attempt to address this issue. In their study, they explore the possibility of generating a synthetic point cloud. To this end, they created a real and virtually identical environment of a sewer pipe and captured both using a PMD Pico Flexx sensor [67], a ToF camera, thereby ensuring the reliability of their virtual framework in comparison to the real setup. Building on this foundational work, Haurum et al. [68] conducted a comprehensive follow-up study in which they developed an extensive dataset of point clouds containing 8500 annotated sewer defects. The dataset categorises defects into three main classes: displacements (misaligned pipe joints), obstructions (such as bricks), and defective rubber rings (compromised sealing rings). The dataset comprises both synthetic and real-world data. Synthetic data were generated through a simulation environment that modelled PVC sewer pipes using a virtual ToF sensor to capture detailed point clouds. Real-world data were collected in a laboratory using actual PVC pipes with induced defects, captured by a Pico Flexx. To reflect practical constraints in sewer inspection, the dataset is divided into training, validation, and testing subsets, with synthetic data primarily used for training and validation and real data for testing, allowing for an effective evaluation of how well models trained on synthetic data perform under real-world conditions. Based on this dataset, Zhou et al. [69] introduced a Transformer-based DL model, TransPCNet, for the classification of sewer defects using 3D point cloud data. The model is specifically developed to classify the four types of sewer conditions: normal, brick, rubber ring, and displacement defects. TransPCNet comprises a feature embedding module that enhances local feature extraction, an attention module derived from Transformer architecture that improves feature learning by focusing on long-range contextual information, and a classification module that employs a weighted smoothing cross-entropy loss function to address data imbalance. The model demonstrated superior performance, achieving a macro average F1-score of 0.70, precision of 0.73, and recall of 0.68, outperforming state-of-the-art methods applied by them to the dataset.

Detecting defects in point clouds, specifically potholes, is also explored by N. Wang et al. [70], who developed a DL model for automated classification and segmentation of 3D sewer pipeline defects. Using a dataset of both real and synthetic point clouds collected from concrete pipes, their method addresses variations in pothole shapes and sizes to ensure robust performance. The authors enhanced the PointNet++ model [71] with residual connections, label smoothing, and advanced training techniques, including AdamW and cosine learning rate decay. Their optimised model achieved a mean intersection over union (mIoU) of 94.15%, outperforming other state-of-the-art methods and effectively advancing pothole detection in sewer pipelines. In another investigation, N. Wang et al. [72] focused on corrosion damage segmentation in concrete drainage pipes using an advanced point Transformer model.

### 3.3. Discussion

In recent years, various methods have been developed to utilise 3D point cloud data from sewage pipes to enable automated damage detection. These methods primarily focus on identifying structural changes in pipe geometry, such as potholes, erosion, deposits, and joint displacements or dislocations. These types of damage can be detected as they create noticeable deviations from the usual pipe surface. Accurately measuring these damages enables highly reliable assessments of their impact and severity on the sewage pipe’s structural health—achieving a level of accuracy that conventional camera-based inspections, which rely on estimates, have yet to reach. However, a major challenge remains before these methods can be applied in routine sewage inspections. Processing, detecting, and measuring damage within 3D point clouds requires either significant technical manual expertise, making the approach more costly for companies and therefore not profitable, or a robust DL model that can autonomously analyse the point cloud. The model, however, must be trained before sufficient data from inspections can be collected, resulting in the need for a synthetic dataset. Such a dataset has already been created, and a DL model was successfully trained on it. However, the dataset is limited to PVC pipes and only three damage types. Therefore, further synthetic data with additional damage classes, including those for concrete sewage pipes, are needed.

## 4. Concept for a Multi-Sensor System and Deep Learning Integration Concept

The quality of data remains a critical weakness in developing automated damage detection and assessment models. In addition to the inaccurate documentation of damage images [33], the data themselves pose challenges. Biswas et al. [34] also highlight the issue of low inter-class variation, which leads to many studies not addressing all types of damage simultaneously, as seen in Table 4. Furthermore, the images captured are often sub-optimal for training due to low camera quality, which is why a large portion of studies apply preprocessing methods (Section 2.2). However, this introduces the risk that preprocessing may be overly optimised for the current dataset, rendering it less effective for newly captured data. As a result, the developed models may not perform as reliably in real-world applications. In this chapter, based on the insights gained from Section 2 and Section 3, a sensor system and damage processing design will be proposed for an inspection robot aimed at providing a data quality high enough to enable automated sewer inspections.

**Figure 5:**
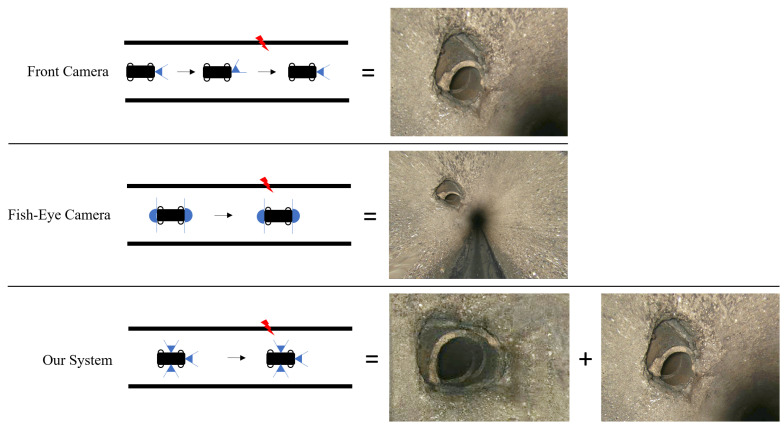
Comparison of three camera systems used in robotic sewer pipe inspection. The first row displays a front-facing camera system, which requires manual adjustment to capture detailed imagery, leading to a restricted field of view. The second row illustrates a fish-eye camera configuration, which provides a wide-angle view but introduces peripheral distortion, resulting in a loss of critical information. The third row showcases the proposed system, which integrates multiple forward- and lateral-facing cameras to deliver undistorted, high-resolution images, ensuring comprehensive coverage of the pipe’s surface.

**Figure 6:**
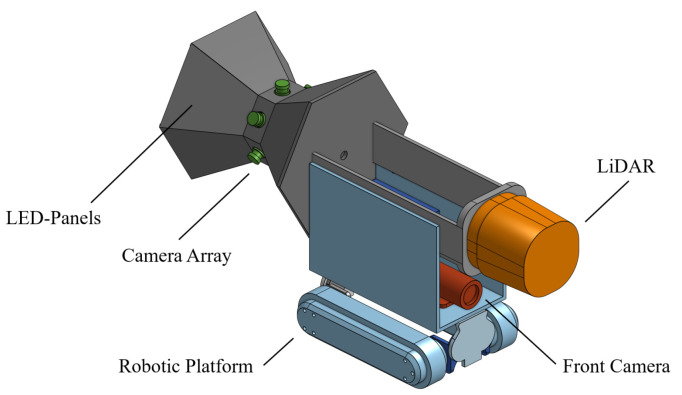
Representation of the robotic platform. The platform is depicted in blue, featuring a LiDAR in orange at the front, a red front-facing camera, and a rear camera array. The camera array (green) is surrounded by LED panels (grey) for optimal illumination.

Our camera configuration is presented in comparison with the currently employed camera systems in Figure 5, while the complete hardware design of our robotic platform is illustrated in Figure 6. Whereas current camera systems often require the camera to be manually tilted towards the damage to capture a complete view, and while fish-eye cameras offer a wide field of view but result in distorted images—particularly at the edges, where the relevant information is primarily located—our system employs multiple cameras with varying orientations to provide a comprehensive, undistorted view of the scene. In addition to utilising a common front camera (Section 4.2), two additional sensor systems will be incorporated, which, although not commonly employed in sewer inspections, hold significant potential for enhanced damage detection. One part is the integration of a LiDAR sensor to improve the detection of geometric sewer damages (Section 4.3). Additionally, a new camera configuration consisting of multiple camera modules arranged in a circular pattern and oriented vertically towards the sewer pipe surface will be employed, facilitating a more uniform and higher-resolution capture of the sewer surface, detailed in Section 4.4.

The damage classes to be detected will be assigned to the respective sensor systems based on the sensor’s optimal detection capabilities. This approach reduces the number of damage classes per sensor, allowing the training of specialised AI models, each focusing on a reduced set of damage types, thereby enhancing specialisation and improving detection accuracy.

### 4.1. Robotic Platform

All sensor systems are integrated into a robotic platform engineered for canal inspections. This platform is a chain-driven, waterproof system with a travel speed of 15 cm/s. The front camera is a high-definition unit, while the camera array consists of six full-HD cameras, each equipped with global shutter technology and synchronised triggering to ensure distortion-free and precise motion capture. To achieve uniform scene illumination, LED panels are strategically arranged around the system.

For 3D mapping and damage detection, a compact sick-multiScan LiDAR [73] will be employed. This scanner provides a ground sampling distance of 0.55 mm/pixel at a pipe diameter of 500 mm, enabling sub-millimetre accuracy. Figure 6 provides an illustration of the robotic platform with the integrated sensor systems; the specifications and expected resolutions for each sensor system at various nominal diameters are detailed in Table 8.

### 4.2. Front Camera: Capturing Internal Damages on 2D Images

The front-facing camera remains an essential component in the inspection robot’s setup. However, the focus in damage detection is shifting from identifying all surface damages on the pipe to exclusively detecting internal damages within the pipe structure. These internal damages—such as collapses, blockages, vermin infestations, and root intrusions, as outlined in Table 9—are critical not only for identifying potential flow blockages but also for ensuring the safe navigation of the inspection robot. While a real-time model would facilitate autonomous navigation, existing models based on the YOLO framework, though optimised for fast computation, are not yet reliable enough to safely detect potential blockages. As a result, the pipe scanning process still requires manual intervention to ensure a scanning without collisions. Damage detection, classification, and severity assessment, however, can be performed in post-processing, allowing the use of architecturally complex and computationally intensive segmentation networks. The best performing models in 2D image segmentation are Mask R-CNN [58], DeepLabv3+ [55], and U-Net [57], which have already been tested and proven functional for damage detection in sewage pipes [22,23,25,26,29]. Although these models do not operate in real time, they provide more stable and precise damage detection and severity assessment. Enhanced camera resolution, improved illumination, and a stable field of view further elevate image quality, making damage features more discernible and ensuring consistency in detected damage features across frames. Additionally, by focusing solely on damage types within the pipe and not on damages at the pipe’s surface, the model’s classification complexity is significantly reduced.

### 4.3. LiDAR: Capturing Geometric Damages in Point Clouds

As discussed in Section 3, the previous studies [60,61,62,64,65] have shown that certain types of pipeline damages can be effectively detected in 3D point clouds. These detectable damages primarily involve geometric changes in the pipeline structure. Damages including holes [60,61], deformations [62,64], obstacles [62], and shifted joints [65] have already been investigated in the previous research to be detectable in point clouds. Beyond these, depressions such as missing masonry, exposed soil, and visible cavities are analogous to potholes. Surface protrusions, including protruding connections, seals, and ground intrusions, are similar to the already investigated obstacles and are also expected to be visible in point clouds. Furthermore, depending on their size, deposits might be recognisable in 3D point clouds. The damages to be detected through point clouds are summarised in Table 10, classified according to the European standard for damage classification. The advantage of point-cloud- over image-based inspection lies in its ability to quantify damages in 3D. While the size of damages in images has traditionally been estimated and is prone to measurement errors, point clouds allow for precise measurement of the extent and severity of the damage through geometric surveying. With the most frequently used pipe diameters between 250 mm and 350 mm, our used LiDAR sensor creates a point cloud with sub-millimetre accuracy, described in Table 8. This enables a measurement preciseness that is not achievable based on images, where diameters and depth of geometric damages have to be estimated. This precise quantification is particularly relevant for the European assessment standard, which requires quantitative parameters for each defect. Accurate measurement is therefore essential for automated inspection. Furthermore, by detecting damages through point clouds, it reduces the complexity for damage detection with image-based sensors, as fewer damages have to be detected.

In the pursuit of realising AI-driven damage detection in point clouds, a sufficiently large dataset of damage examples is indispensable, presenting the greatest challenge in its development. Collecting enough data from real-world channels is hardly feasible, which is why, as in [68], an artificially generated dataset must be utilised. That dataset currently includes only damages for PVC pipes, making it necessary to create a dataset for concrete pipes. A second challenge lies in accurately differentiating between various types of damage. While defective masonry is relatively easy to distinguish from a protruding connection, the similarity to a visible cavity is much greater, making accurate differentiation more difficult. To ensure reliable distinction, it is advisable to fuse the point cloud data with images captured by the camera array, creating a RGB-Depth map of the pipes surface, which further enable analysis by combining colour and depth information. To detect damages within the point clouds, two approaches can be adopted. The first involves using RANSAC or DBSCAN to extract anomalies from the normal pipe surface, which reduces the complexity for the subsequent DL model. Alternatively, the point clouds can be directly fed into the network, which provides additional contextual information for the model but incurs higher computational costs. DL models such as point Transformer [74] and KPConv [75] are particularly effective when RGB data is integrated into the point cloud, as this enhances the model’s ability to discern damage.

### 4.4. Camera Array: Capturing Surface Damages on a 2D Surface Map

Damages that do not result in significant geometric changes are still best detected through images. However, the current image quality is sub-optimal in several respects. First, most damages occur on the surface of the pipeline, which requires an operator to rotate the camera head towards the damage, a process that is not feasible for automated inspections. Second, the field of view of the camera is limited. This reduces the capability of capturing context information around the damage, which is necessary for the reliance of the network, and larger damages such as long cracks cannot be captured completely in the image, which prohibits a reliable quantification of the damage. Ultimately, in current inspections, only images of damages were taken, not of the normal pipe surface. Therefore there is a scarcity of images without damages to properly train the network.

One approach to consistently capture the whole surface of the pipe is using fish-eye cameras [76], which captures both the sidewalls and the centre of the pipe. This eliminates the need for manual swivelling, allowing for autonomous inspection. However, a significant portion of the fish-eye camera’s image is focused on the centre of the pipe, which, due to poor lighting and increasing distance from the damage, is unusable. The peripheral area of the image is therefore of utmost importance but is often cropped [77,78] during the necessary conversion into peripheral images [76]. This makes the application of fish-eye cameras sub-optimal due to the relevant image area being located at the edge of the fish-eye lens.

**Figure 7:**
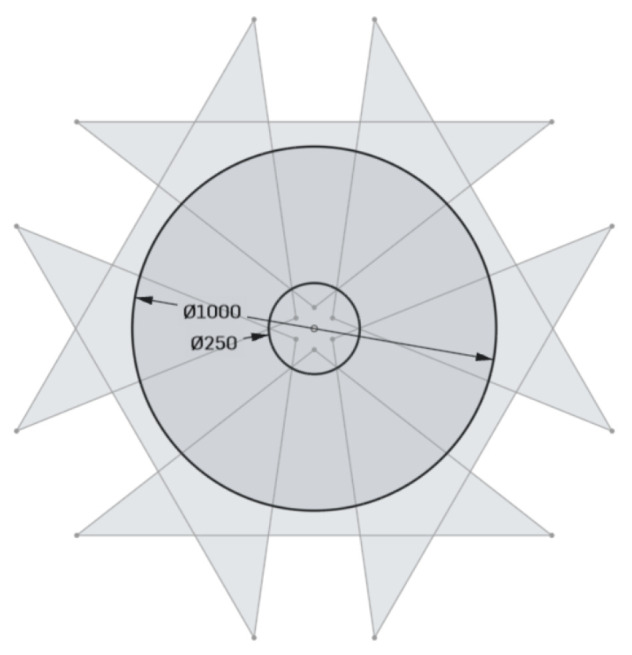
Cross-sectional view of camera array with six cameras with overlapping fields of view beginning at a pipe diameter of 250 mm, ensuring uniform coverage of the entire pipe surface.

To achieve a uniform, high-resolution image of the pipeline surface with consistent lighting and minimal distortion, our approach draws inspiration from current systems employed in tunnel inspections [79]. These systems utilise a ring of cameras combined with LEDs for proper lighting, mounted onto a vehicle, such as a car or train. As the vehicle moves through the tunnel, images are captured continuously. Similarly, our setup employs six cameras, as illustrated in Figure 7, arranged perpendicularly to the pipe’s surface with overlapping fields of view. Each camera features a full-HD resolution, which enables scanning the surface with sub-millimetre accuracy for pipe diameters up to 2000 mm, as described in Table 8. To ensure consistent illumination, a ring of LEDs is incorporated, as shown in Figure 6. The captured images overlap both around the pipe’s circumference and along its length. These images are subsequently stitched together to create a high-resolution, evenly illuminated 2D map of the entire pipeline surface. This approach offers several advantages. First, the DL models benefit from additional contextual information surrounding the damage. With a wider context, the models can better distinguish the damage from the normal pipe surface, improving their ability to accurately identify and classify defects. Secondly, the inclusion of images showing undamaged sections of the pipe significantly enriches the dataset used to train the DL models. Previously, the dataset solely contained images of damaged areas, leaving the models without a reference for what a normal, intact pipe surface should look like. By incorporating these images of undamaged pipe surfaces, the models learn to recognise the features of a standard pipe, improving their ability to detect deviations and reducing the likelihood of misclassifying normal surfaces as defective. Lastly, the high resolution of the images, combined with the consistent distance of the cameras to the pipe surface, enhances both the detection and quantification of damages. The improved feature visibility enables better segmentation, while the consistent distance ensures accurate measurement of damage dimensions with millimetre accuracy—significantly advancing the inspection process compared to the prior reliance on estimation-based assessments by human inspectors working with inconsistent images.

The damages targeted for detection on the 2D image map include surface defects such as defective connections, linings, repairs, welds, infiltration, visible ground, and cavities, as summarised in Table 11. For certain types of damage, such as defective connections and visible ground, which involve geometric changes to the pipe’s surface, integrating depth information from the 3D point cloud captured by the LiDAR sensor can enhance the model’s detection capabilities. Since no dedicated dataset currently exists for this specific application, and images from current sewage inspections differ significantly from the desired data format, the training of the DL models must initially rely on images from other domains. Potential sources include datasets from tunnel inspections or sewage pipe inspections conducted with fish-eye cameras, which can also generate a 2D map of the pipe’s surface—albeit not at the same resolution achievable with the proposed system. To effectively detect larger damages, the 2D map of the pipe surface should be provided to the DL models in sufficiently large sections. This requires a segmentation network capable of processing large-scale images. Advanced architectures, such as the hybrid task cascade (HTC) [80] or a Mask R-CNN [58] with a Swin Transformer [81] backbone, are well-suited for this task. These models combine precise instance-level segmentation with robust contextual understanding, making them particularly effective for analysing large-scale, high-resolution images. However, due to their substantial computational demands, such models are better suited for post-processing tasks rather than real-time applications.

### 4.5. Discussion

The proposed sensor and processing concept for a new robotic platform is designed to significantly enhance data quality in future sewage inspections. By improving data quality, damage features are expected to become more clearly visible, thereby enhancing the performance of DL models and advancing fully automated damage inspection. To reduce the complexity of the current approach—which relies on a single front-facing camera to differentiate between approximately 20 damage classes—the pipe environment is now captured using multiple sensors. In addition to the front-facing camera, which captures damage within the pipe, a ring of cameras is positioned perpendicularly to the pipe surface. These cameras capture high-resolution images with consistent illumination and stable viewing angles. The overlapping images can then be stitched together to create an unrolled 2D map of the pipe surface, facilitating the detection and measurement of larger damages, such as extensive cracks, that exceed a single image’s field of view. Furthermore, a LiDAR sensor generates a 3D point cloud of the pipe, enabling more precise detection and quantification of geometric damages than is possible with images alone. By integrating these new sensor setups, key limitations of current systems—such as low-resolution and unevenly illuminated images, inconsistent camera orientation, and the limited detectability of geometric damages—are effectively addressed, as summarised in Table 12.

Using DL models to detect, classify, and quantify damages in images and point clouds has been extensively researched in recent years. For front-facing camera images, segmentation models such as Mask R-CNN, DeepLabv3+, and U-Net have been tested and demonstrated their effectiveness [22,23,25,26,29]. However, the 2D surface map created by the camera array requires segmentation models capable of processing large-scale images, such as hybrid task cascade (HTC) or Mask R-CNN with a Swin Transformer backbone. For 3D point clouds, state-of-the-art segmentation models include PointNet++ and KPConv. These models can potentially incorporate additional RGB data from the camera array images, which may further enhance detection performance by providing valuable texture and colour information alongside depth data. PointNet++ has already been applied to damage segmentation in sewage pipes with excellent results [70]. If the point clouds are found to be excessively large for efficient processing, a potential preprocessing step is to reduce the data size by removing the normal pipe surface using algorithms such as DBSCAN or RANSAC. Table 13 summarises the most suitable models for each data source and highlights the anticipated challenges in their development.

Both the camera array and the LiDAR sensor produce high-resolution data outputs that are not yet widely utilised in sewage pipe inspection. Consequently, developing DL models for these sensors requires either adapting images from other domains or creating a synthetic dataset. Despite the additional effort required to integrate these systems into sewage inspections, their inclusion is expected to significantly enhance inspection quality. Both methods capture the pipe surface at sub-millimetre resolution, enabling damage quantification with millimetre accuracy. Compared to the previous method of manual damage size estimation—based on low-resolution images and performed by human inspectors at a centimetre scale—these sensors provide a precise, automated, and reliable approach for detecting and quantifying damages in sewage pipes.

Testing this prototype will raise several questions. For instance, as the cameras face the surface directly, certain damages could be overlooked, such as spalling along the sides of inlet connections, which would be visible in a side view. This issue may require additional side-facing cameras or supplementary use of the front camera. Moreover, the LiDAR scanner presents a trade-off: either the surface is scanned as a high-resolution point cloud—at the cost of longer scanning and inspection time—or in a faster scan, resulting in a sparser point cloud with the risk of missing finer details. Finally, generating an accurate point cloud requires a reliable SLAM algorithm to correct for measurement errors caused by the movement of the robotic platform. This poses a significant challenge, given the limited features within the pipe and the availability of only two global reference points at the pipe’s start and end.

## 5. Conclusions

In this study, we conducted a comprehensive review of state-of-the-art methods in automated sewer inspection, examining both image-based techniques and point cloud approaches for automated damage detection. Image-based models, the predominant choice for sewer inspection, largely rely on data from previous manual inspections. Broadly, automated sewage pipe inspection can be categorised into three methodological approaches: image classification, object detection, and segmentation. Much of the research has concentrated on damage-type detection, often emphasising computational efficiency to enable real-time application and support human inspection. Segmentation-based methods, which delineate damage regions and assess damage severity, aim toward a fully autonomous network for post-inspection analysis.

However, significant challenges persist, largely due to poor dataset quality. Issues such as mislabelled images from manual inspections, low-resolution images with inadequate lighting, incomplete damage capture due to limited field of view, and low inter-class variability across more than 20 damage classes impede the development of a model capable of fully automated inspection without human intervention. While many researchers have achieved promising results, these studies generally focus on a subset of damage types, leaving questions about the model’s effectiveness on comprehensive datasets unanswered. Moreover, comparability across approaches is severely limited, as each researcher uses distinct datasets with different types of damage considered, further complicating cross-study evaluations.

Beyond image-based techniques, point clouds offer a promising alternative for sewer damage detection. Although not yet utilised in current inspections, point clouds hold significant potential for detecting geometric abnormalities not readily visible in images. Preliminary field studies indicate that point clouds can facilitate more accurate damage detection and quantification than image-based methods. The main obstacle here is the lack of sufficient training data, a critical requirement for the development of robust DL models. While early attempts have been made to create synthetic datasets for point-cloud-based DL models, the variety of damage types in these datasets remains limited.

To address the limitations of inadequate data quality, we propose a sensor system concept that captures both images and point clouds in a standardised manner, creating a dataset suitable for the development of DL models. The system includes a front-facing camera and multiple cameras arranged in a ring configuration, oriented directly toward the pipe surface. These cameras capture images with millimetre resolution under consistent illumination. By stitching these images together, a 2D map of the unrolled pipe surface can be generated, enabling the detection of damage areas larger than a single camera’s field of view. This approach eliminates the need to tilt the front camera toward the damage, facilitating a standardised view of each defect and reducing human intervention in inspections. Additionally, a LiDAR sensor generates a 3D point cloud with millimetre accuracy, enabling the detection and quantification of geometric defects. This reduces the complexity required of image-based DL models by excluding geometrically detected defects.

In this work, damage types were allocated across the three sensor systems, allowing each defect to be captured with the optimal sensor and thereby reducing the complexity of each DL model. With the combined use of the camera array and LiDAR sensor, the pipe surface can be captured on a millimetre scale, enabling precise, automated damage measurement—a significant improvement over manual size estimation by inspection workers, which was previously performed at a centimetre scale.

Nevertheless, several challenges remain for both the sensor system and the DL models. For the side-facing cameras, certain defects may be obscured by inlet pipes, which were previously visible in a side view. For the LiDAR sensor, a balance must be achieved between scan speed and point cloud density, as lower-density scans risk missing defects. Finally a robust SLAM algorithm is necessary to minimise measurement errors, which can be challenging in confined pipe environments. The development of DL models for each sensor system is hindered by limited data availability. However, datasets from fisheye camera inspections could be generated, and existing datasets for individual damage types, such as cracks, could serve to pre-train models that can then be fine-tuned with newly collected data. To address the lack of training data, it would be helpful to create a synthetic dataset of concrete sewer pipes with typical damage types for point-cloud-based DL models. 

## Figures and Tables

**Table 1:** Damage categories in the European pipe inspection norm DIN EN 13508-2 [6] with their required damage characterisation and further information.

Damage	Code	Characterisation	Position in Pipe ^1^	Additional Information
Deformation	BAA		X	CSR ^2^
Cracking	BAB	Crack type, crack pattern	X	Crack width
Pipe break/collapse	BAC	Break/collapse	X	Length
Defective masonry	BAD	Shifted/sagged	X	Depth of sagging
Missing mortar	BAE			Depth of hole
Surface damage	BAF	Severity of damage, cause	X	
Protruding connection	BAG		X	CSR
Defective connection	BAH	Type of damage	X	
Protruding seal	BAI	Seal type, broken/shifted	X	CSR
Shifted joint	BAJ	Type and length/severity of shift		
Defective lining	BAK	Type of material defect, progression	X	
Defective repair	BAL	Type of defect, progression	X	
Defective weld	BAM	Progression	X	
Porous pipe	BAN		X	
Visible ground	BAO		X	
Visible cavity	BAP		X	
Roots	BBA	Root type	X	CSR
Adhering substances	BBB	Substance type	X	CSR
Deposits	BBC	Material type	X	CSR
Soil intrusion	BBD	Soil type	X	CSR
Other obstacles	BBE	Masonry, object, foreign line crossing	X	CSR
Infiltration	BBF	Water flow rate	X	
Vermin	BBH	Animal type		

Note ^1,2^: CSR = Cross-sectional reduction (%): Indicates the percentage of the pipe’s cross-sectional area that is obstructed or reduced due to damage or deposits. Position in Pipe: Requires the specific location along the pipe’s circumference.

**Table 2:** Overview of public sewer inspection datasets.

Dataset Name	Year	Data Type	Size	Defect Classes	Tasks	Region
Sewer-ML [30]	2021	Images	1.3 million images	18 classes	Multi-label defect classification	Denmark
VideoPipe: QV-Pipe [31]	2022	Short video clips	9601 clips (>55 h)	17 classes	Video defect classification	Shenzhen, China
VideoPipe: CCTV-Pipe [31]	2022	Long untrimmed videos	575 videos (87 h)	16 classes	Temporal defect localisation	Shenzhen, China
Storm Drain Dataset [32]	2021	Images	1000 images	6 classes	Damage detection and classification	Not specified
Pipe-SOLO [27]	2022	Images	3888 images	6 classes	Damage detection and segmentation	Seoul, South Korea

**Table 3:** Overview of evaluation metrics and their explanations.

Metric	Explanation
Accuracy (Acc)	The percentage of correctly classified instances in the dataset.
F1-Score (F1)	The harmonic mean of precision and recall, balancing false positives and false negatives.
Mean Average Precision (mAP)	The average precision across all recall levels, often used in multi-class classification and object detection.
mAP@0.5:0.95	The mean of mAP calculated at IoU thresholds ranging from e.g., 0.5 to 0.95, assessing both detection and localisation accuracy.
IDF1 (Identity F1 Score)	A metric for multi-object tracking that combines precision and recall, with a focus on maintaining identity consistency over time.
Top-1 Accuracy (Top-1 Acc)	The proportion of instances where the highest-confidence prediction matches the correct class.
IoU@0.5	Intersection over Union at a 0.5 threshold, measuring the overlap between predicted and ground truth instances.
Mean IoU (mIoU)	The average IoU across all classes, commonly used to evaluate performance in semantic segmentation.
Mean Pixel Accuracy (mPA)	The average pixel-level accuracy across all classes in segmentation tasks, providing a detailed assessment of performance.

**Table 4:** Overview of covered damages in automated sewer inspection papers, illustrating the focused nature of the existing studies, which predominantly address only a limited number of damage categories. Common defects like cracked joints, spalls, and breaks/collapse are extensively studied, whereas rare defects are under-represented, probably due to missing data availability.

Damage Category/Author	[34]	[21]	[38]	[39]	[19]	[33]	[35]	[16]	[23]	[18]	[24]	[40]	[14]	[12]	[37]	[17]	[20]	[41]	[36]	[13]	[27]	[29]	[42]	[22]	[43]	[25]	[44]	[45]	[26]	[15]	[28]
Cracking	X	X	X	X	X	X	X	X	X	X		X	X	X		X	X	X		X	X		X	X		X	X	X	X		
Shifted joint	X	X	X	X	X	X	X	X	X	X	X		X		X	X	X		X		X	X	X	X	X					X	
Roots	X	X	X	X		X	X		X	X	X	X			X		X	X		X		X	X	X	X		X			X	
Pipe break/collapse	X	X	X	X	X	X	X	X	X			X	X	X	X	X		X			X	X				X			X		
Protruding connection	X	X	X	X	X		X	X	X		X		X	X	X	X		X			X						X				
Other obstacles	X	X	X	X	X	X			X	X	X			X	X		X		X				X	X						X	
Surface damage	X	X	X	X		X	X	X	X		X		X			X			X		X					X		X			
Deposits	X	X	X	X	X	X	X				X	X		X		X	X	X		X		X			X						X
Deformation	X	X	X	X	X	X				X				X			X					X			X			X	X		
Defective connection	X	X	X	X	X		X	X					X									X				X					
Infiltration	X	X	X			X						X							X	X											
Adhering substances	X	X		X				X		X	X																				X
Protruding seal	X	X			X	X		X							X																
Visible cavity	X								X	X		X						X													
Defective lining	X			X	X																										
Visible ground	X		X									X																			
Porous pipe	X																														
Soil intrusion	X																														
Defective repair	X																														
Defective weld																															
Vermin																															
Defective masonry																															
Missing mortar																															

**Table 5:** Overview of papers on defect classification in images from sewage pipe inspections between 2021–2024.

Author	Ref	Year	Dataset	Preprocessing	Base Algorithm	Evaluation	Remarks
Biswas et al.	[34]	2023	156 k	-	HRNet, ResNet-152, MobNetV3L, DenseNet-264, IncV4, EffNet	Acc: 90.13%, F1: 0.89	Comparison between several models
X. Wang et al.	[33]	2023	Sewer-ML [30]	-	CNN	Acc: 82%, F1: 0.81	Binary classification (defect/ no defect)
Hu et al.	[38]	2024	142 h of video	-	Evidential deep learning (EDL)	mAP: 62.56%	Trustworthy classification

**Table 6:** Overview of papers on defect detection in images from sewage pipe inspections between 2021–2024.

Author	Ref	Year	Dataset ^*^ (Images)	Preprocessing	Base Algorithm	Evaluation	Remarks
Tan et al.	[13]	2021	1260/3 k	deleting similar images	YOLOV3	mAP: 92.0%	Adaptive anchor box calculation integrated into training
Yin et al.	[41]	2021	3.6 k	-	YOLOV3, Video interpretation algorithm	F1: 0.75	Report file about damages in pipe
Oh et al.	[19]	2022	4.5 k/12.5 k	text in image enhanced	YOLOV5	mAP: 75.8%	Text recognition for information gain
Situ et al.	[42]	2023	1.2 k	-	YOLOV2 combined with CNNs	mAP: 71.0%	Transfer learning using pretrained CNNs
T. Wang et al.	[37]	2023	2.1 k	HSV Enhancement	YOLOV5	mAP@0.5:0.95: 48.7%	Lightweight design
Situ et al.	[15]	2024	2 k/-	Colour transformation, defogging	YOLOV5	mAP: 91.8%	Lightweight model optimised for speed
Huang et al.	[17]	2024	28 k/-	-	YOLOV5l6	mAP@0.5:0.95: 68.5%	Enhanced feature extraction
Zhao et al.	[18]	2024	1.5 k/-	low-quality images removed	YOLOV5	mAP: 84.0%	Lightweight design
Dang et al.	[14]	2021	38 k/115 k	blurring, DHE, noise reduction	fine-tuned VGG 19	Acc: 97.6%	Text recognition for information gain
D. Li et al.	[12]	2021	10 k/20 k	DHE, noise reduction	Strengthened region proposal network, fine-grained classification network	mAP: 71.3%	Severity grading
M. Wang et al.	[44]	2021	10 k (107 tracked damages)	-	DL and Kalman filter	IDF1: 57.4%	Tracking and re-identification of damages
Ma et al.	[36]	2021	14 k	brightness, noise, contrast, saturation, sharpness	fusion CNN	F1: 0.955	Synthetic data creation
Guo et al.	[45]	2022	7 k	-	Faster-RCNN with P-EFPN	mAP: 75.39%	Focus on edge region defects
Dang et al.	[35]	2022	47 k	DHE, contrast enhancement, denoising	Detection Transformer	mAP: 60.2%	Severity grading
Shen et al.	[43]	2023	4 k	-	improved SSD	mAP: 92.2%	Enhanced small defect detection
Y. Li et al.	[16]	2023	4.4 k/14 k	-	Pipe-VFNet	mAP: 73.4%	Enhanced feature extraction
Yu et al.	[20]	2024	6.7 k/-	denoising, brightness, adjustment, sharpening	Swin Transformer	mAP%@0.5: 78.6%	Model fusion for enhanced detection
Yang et al.	[21]	2024	2.3 k/7.1 k	-	Collaborative localisation module (CLM)	Top-1-Acc: 69.76%	Weakly supervised detection using image-level labels
Yusuf et al.	[28]	2024	2 k/50 k	brightness reduction	fine-tuned VGG 19	Acc: 96.0%	Maximum precision in encrustation detection

* Note: In the column Datasets, values presented as two numbers separated by a ‘/’ represent the dataset sizes before and after data augmentation, respectively. A ‘-’ denotes that no information is available regarding dataset sizes either before or after augmentation.

**Table 7:** Overview of papers on defect segmentation in images from sewage pipe inspections between 2021–2024.

Author	Ref	Year	Dataset ^*^ (Images)	Preprocessing	Base Algorithm	Evaluation	Remarks
M. Wang et al.	[40]	2021	3000	contrast enhancement	DilaSeg-CRF	F1: 88.99%	Severity assessment (pipe-cross section)
Zhou et al.	[22]	2022	600/44 k	-	DeepLabv3+	mIoU: 0.53, F1: 0.55	Severity rating
He et al.	[24]	2022	700/3.6 k	contrast enhancement	SegNet	mIoU: 0.67	Multi-defect per image
Fang et al.	[26]	2022	1.7 k/-	-	Mask R-CNN	IoU@0.5: 92.7%	Creation of 3D models
Y. Li et al.	[27]	2022	3.9 k/9.2 k	dehazing	Pipe-SOLO	mAP: 59.3%	Dataset published
Rayhana et al.	[25]	2023	5.6 k/-	-	MaskC-RCNN	F1: 0.87	Robust detection
Dang et al.	[23]	2023	3.7 k/11 k	contrast enhancement, dehazing	DeepLabV3+	mPA: 97.9%, mIoU: 0.689	Severity rating
M. Li et al.	[29]	2024	1.7 k/-	-	U-Net	mIoU: 0.72	Severity rating

* Note: In the column Dataset, values presented as two numbers separated by a ‘/’ represent the dataset sizes before and after data augmentation, respectively. A ‘-’ denotes that no information is available regarding dataset sizes either before or after augmentation.

**Table 8:** Specifications of the sensor systems used and their expected measurement resolutions based on the nominal diameter.

			Nominal Diameter (DN) [mm]			
**Description**	**Unit**	**Value**	**250**	**500**	**1000**	**2000**
Front camera						
Resolution Width	[Pixel]	1920				
Resolution Height	[Pixel]	1080				
Camera array						
Number of cameras		6				
Image format width	[Pixel]	1920				
Image format height	[Pixel]	1200				
Exposure time	[ms]	1				
Camera FPS	[Hz]	30				
Resolution width	[mm/Pixel]		0.082	0.164	0.327	0.654
Resolution height	[mm/Pixel]		0.084	0.173	0.347	0.692
Distance travelled per image	[m]	0.005				
Image blur at Vmax	[mm]	0.15				
LiDAR						
Sampling frequency	[Hz]	20				
Opening angle in travel direction	[°]	65				
Scan layers	[#]	2				
Angular resolution	[°]	0.125				
Spatial resolution in travel direction	[mm]	0.75				
Spatial resolution radial	[mm]		0.27	0.55	1.09	2.18
Scan rate	[cm/s]	15				

**Table 9:** Damages to be detected based on images captured by the front camera, along with their corresponding codes from DIN EN 13508-2 [6].

Code	Description
BAC	Pipe break/collapse
BAO	Visible ground
BBA	Roots
BBD	Soil intrusion
BBE	Other obstacles
BBH	Vermin

**Table 10:** Damages to be detected by point-cloud-based methods along with their corresponding codes from DIN EN 13508-2 [6].

Code	Description
BAA	Deformation
BAC	Pipe break/collapse
BAD	Defective masonry
BAG	Protruding connection
BAI	Protruding seal
BAJ	Shifted joint
BAO	Visible ground
BAP	Visible cavity
BBC	Deposits
BBD	Soil intrusion
BBE	Other obstacles

**Table 11:** Damages to be detected based on images captured by the camera array, along with their corresponding codes from DIN EN 13508-2 [6].

Code	Description
BAB	Cracking
BAF	Surface damage
BAH	Defective connection
BAK	Defective lining
BAL	Defective repair
BAM	Defective weld
BAO	Visible ground
BAP	Visible cavity
BBB	Adhering substances
BBF	Infiltration

**Table 12:** Limitations of existing inspection systems and proposed improvements in sensor design.

Identified Limitation	Proposed Improvement
Low-resolution image quality	Implementation of a minimum full-HD resolution for the front camera and a camera array capable of detecting millimetre surface details
Inconsistent camera orientation towards damage features	Consistent imaging of damage features using a camera array aligned perpendicular to the pipe surface for surface damage, combined with a fixed front camera for consistent detection of blockages, thereby enhancing damage comparability
Limited coverage of the pipe due to manual control of camera direction	Complete coverage of the pipe surface through the camera array
Lack of context in damage images due to a narrow field of view	Full 2D-map of surface for context-based detection of damages
Inhomogeneous illumination (over- and underexposure)	Integration of a uniform illumination system with a ring of LED panels to ensure consistent lighting conditions
Limited acquisition and detection of geometric damages in 2D imagery	Incorporation of 3D data acquisition through a LiDAR sensor, resulting in an RGB-depth map when fused with 2D images from the camera array, enabling accurate detection and quantification of geometric deformations

**Table 13:** Proposed DL models and algorithms for the different sensors in our proposed sensor concept and expected challenges.

Data Source	Proposed DL Models	Expected Challenges
Front camera images	Segmentation models, e.g., Mask R-CNN [58] DeepLabv3+ [55] U-Net [57]	Difficulty in feature extraction from low-contrast areas
2D surface map derived by camera array	Segmentation on large-scale images, e.g., Hybrid Task Cascade [80] Swin Transformer with Mask R-CNN [58,81]	Lack of data for damage images captured perpendicular to the surface
3D point clouds + optional RGB information	Preprocessing of point cloud, e.g., DBSCAN [63] RANSAC [82] Segmentation on 3D point clouds + RGB, e.g., PointNet++ [71] KPConv [75]	Loss of details in sparse point clouds High computational costs for dense point clouds Lack of real-world data

## Data Availability

The data presented in this study are available on request from the corresponding author.

## Appendix A. Overview of Published Papers in the Last 4 Years and Their Goals

**Table A1:** Overview of damage detection, classification, and segmentation approaches used in the reviewed papers.

Author	Ref	Year	Damage		
			Detection	Classification	Segmentation
M. Wang et al.	[40]	2021	X	X	X
M. Wang et al.	[44]	2021	X	X	
D. Li et al.	[12]	2021	X	X	
Yin et al.	[41]	2021	X	X	
Ma et al.	[36]	2021	X	X	
Tan et al.	[13]	2021	X	X	X
Dang et al.	[14]	2021	X	X	
Dang et al.	[35]	2022	X	X	
He et al.	[24]	2022		X	X
Y. Li et al.	[27]	2022		X	X
Fang et al.	[26]	2022	X	X	X
Oh et al.	[19]	2022	X	X	
Zhou et al.	[22]	2022		X	X
Guo et al.	[45]	2022	X	X	
Rayhana et al.	[25]	2023	X	X	X
Shen et al.	[43]	2023	X	X	
Dang et al.	[23]	2023	X	X	X
Situ et al.	[42]	2023	X	X	
Y. Li et al.	[16]	2023	X	X	
X. Wang et al.	[33]	2023		X	
T. Wang et al.	[37]	2023	X	X	
Chen et al.	[39]	2023		X	
Biswas et al.	[34]	2023		X	
Situ et al.	[15]	2024	X	X	
Huang et al.	[17]	2024	X	X	
Zhao et al.	[18]	2024	X	X	
Yu et al.	[20]	2024	X	X	
Hu et al.	[38]	2024		X	
Yang et al.	[21]	2024	X	X	
Yusuf et al.	[28]	2024	X	X	
M. Li et al.	[29]	2024		X	X
